# Occupationally Acquired American Cutaneous Leishmaniasis

**DOI:** 10.1155/2012/279517

**Published:** 2012-11-28

**Authors:** Maria Edileuza Felinto de Brito, Maria Sandra Andrade, Éricka Lima de Almeida, Ângela Cristina Rapela Medeiros, Roberto Pereira Werkhäuser, Ana Isabele Freitas de Araújo, Sinval Pinto Brandão-Filho, Alzira Maria Paiva de Almeida, Eduardo Henrique Gomes Rodrigues

**Affiliations:** ^1^Centro de Pesquisas Aggeu Magalhães, FIOCRUZ-PE, Campus da Universidade Federal de Pernambuco s/n, Cidade Universitária, 50.670-901 Recife, PE, Brazil; ^2^Centro de Ciências Biológicas, Universidade Federal de Pernambuco s/n, Cidade Universitária, 50.670-901 Recife, PE, Brazil; ^3^Departamentos de Enfermagem, Universidade de Pernambuco, Rua Arnóbio Marques 310, Santo Amaro, 50.100-130 Recife, PE, Brazil; ^4^Departamentos de Dermatologia, Universidade de Pernambuco, Rua Arnóbio Marques 310, Santo Amaro, 50.100-130 Recife, PE, Brazil

## Abstract

We report two occupationally acquired cases of American cutaneous leishmaniasis (ACL): one accidental laboratory autoinoculation by contaminated needlestick while handling an ACL lesion sample, and one acquired during field studies on bird biology. Polymerase chain reaction (PCR) assays of patient lesions were positive for *Leishmania*, subgenus *Viannia*. One isolate was obtained by culture (from patient 2 biopsy samples) and characterized as *Leishmania* (*Viannia*) *naiffi* through an indirect immunofluorescence assay (IFA) with species-specific monoclonal antibodies (mAbs) and by multilocus enzyme electrophoresis (MLEE). Patients were successfully treated with N-methyl-glucamine. These two cases highlight the potential risks of laboratory and field work and the need to comply with strict biosafety procedures in daily routines. The swab collection method, coupled with PCR detection, has greatly improved ACL laboratory diagnosis.

## 1. Introduction

Health professionals such as physicians, nurses, and laboratory workers and researchers and students in the biological sciences are at risk of a number of occupational infections [1]. Accidents involving contaminated “sharps” are, in general, extremely dangerous due to the high likelihood of infectious agent transmission. Exposure to blood-borne pathogens represents an especially serious risk. Needlestick and sharps contamination accidents involving at least 20 different pathogens, most commonly hepatitis B and C or human immunodeficiency virus (HIV), have been reported [1]. Some occupational accidents with *Leishmania* spp. related to percutaneous injuries, contaminated animal/culture handling, or lesion sample collection have also been reported [2].

ACL diagnosis is based on clinical, epidemiological, and laboratorial criteria. However, clinical diagnosis is often difficult due to the varied presentation of the disease, and the epidemiological criteria may go unnoticed because clinicians are not aware of the existence or nature of the disease [3, 4].

Laboratory diagnosis includes the direct identification of amastigotes through direct examinations of stained smears from imprints or histological sections, isolation of promastigotes by culture, and immune-based methods (e.g., ELISA, Western blot, indirect immunofluorescence (IFAT), and delayed hypersensitivity or Montenegro intradermoreaction (MIDR), [5, 6].

Laboratory procedures may be hindered by the scarcity of amastigotes in the wounds, especially in late stages of the disease and secondary infections, or by the low sensitivity and cross reactivity of serological tests. Furthermore, the MIDR test does not distinguish between current and past infections, and it is usually negative in patients with the diffuse form of the disease or who are immunosuppressed [5].

Polymerase-chain-reaction (PCR-) based methods provide high sensitivity and specificity, especially when performed in biopsy samples [7, 8]. However, the collection of biopsy material is painful, requiring anesthesia, and it can only be performed by physicians in nosocomial environment, which may increase the risk of iatrogenic bacterial infections. Sample collection using a cotton swab is noninvasive, simple, rapid, and easy, does not require hospitalization, and is associated with low infection risk and patient discomfort [9].

Here, we report a successful combination of non-invasive sample collection using the “swab” method and PCR for the diagnosis of ACL in two occupationally acquired cases.

## 2. Case Reports

### 2.1. Case 1

Case 1 is a 60-year-old female residing in Recife, PE, Brazil and who has worked for more than 20 years in an ACL research laboratory.

**Figure 1:**
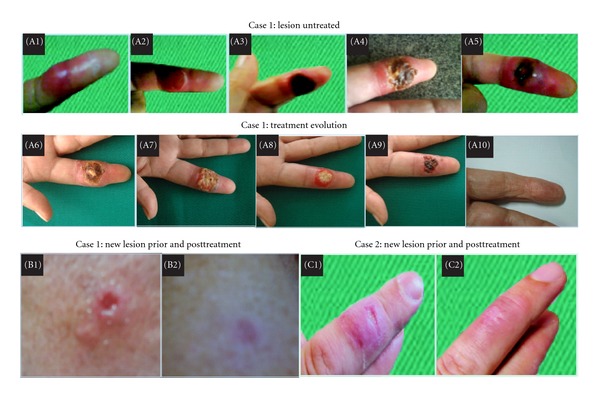
(A1): papule on 2nd left finger. (A2)–(A5): four months evolution prior to treatment. (A6)–(A10): lesion healing. (B1): reoccurrence, new lesion detection thirty days post treatment. (B2): healed lesion. (C1): lesion on 3rd right finger. (C2): healed lesion.

Five weeks after a needlestick accidental auto-inoculation while processing a sample from a patient ACL lesion, she developed a papule at the point of inoculation on the 2nd finger of the left hand. The papule evolved into an ulcer with high edges, measuring 2.0 × 3.0 mm, with production of exudates (Figure 1, (A1)–(A5)), adjacent lymphangitis, and left-axillary ganglion swelling.

X-rays and magnetic resonance imaging revealed no bone degradation (data not shown). Exudates culture revealed *Acinetobacter baumannii* and *Enterococcus faecalis*. Tigecycline was administered but did not promote wound healing. A lesion sample was collected using sterilized swab, and PCR testing was positive for *Leishmania,* subgenus *Viannia *[10]. Two treatment cycles with N-methyl-glucamine (15 mg Sb^+v^/kg/day) for twenty days were necessary for complete healing. The lesion began to heal at day 15 and completed by day 18 (Figure 1, (A6)–(A10)). However, one month later, a new lesion measuring 1.0 × 1.0 mm presented in the right forearm (Figure 1, (B1)). Swab collection and PCR ACL detection again returned a positive result for *Leishmania,* subgenus *Viannia* [10]. Complete remission of the lesion and clinical resolution were achieved at day 20 of the second treatment cycle (Figure 1, (B2)). During the treatments, the patient reported symptoms that may have been related to side effects of the medication, such as asthenia and arthralgia; nevertheless, the treatments were concluded successfully.

### 2.2. Case 2

A 35-year-old male doctoral student noticed the emergence of an ulcerated lesion with high edges, measuring 2.0 × 1.5 mm (Figure 1, (C1)) with exudates on the 3rd finger of the right hand, suggestive of ACL. The lesion was first noted two months after fieldwork capturing birds in the forests in Paranati, Mato Grosso, Brazil.

Routine clinical and laboratory examinations comprising immunological and parasitological tests were carried out in the SRL (Leishmaniasis Reference Laboratory, FIOCRUZ-PE, Recife, PE, Brazil), and lesion samples were collected both by biopsy and the swab method. Optical microscopy showed amastigote parasites on Giemsa-stained smears; MIDR was positive within 48 h, and biopsy sample culturing in NNN/Schneider media for 5 days at 26°C enabled parasite isolation. The isolate was characterized as *Leishmania (Viannia) naiffi* through IFA with species-specific monoclonal antibodies (mAbs) [11] and by multilocus enzyme electrophoresis (MLEE) [12]. PCR reactions from biopsy and swab samples were both positive for *Leishmania,* subgenus *Viannia* [10].

The patient was treated with N-methyl-glucamine (15 mg Sb^+5^/kg/day) for 20 days. Injury healing began at day 11 and completed after the completion of treatment (Figure 1, (C2)).

The patient had some complaints of asthenia and arthralgia during the treatment that were related to side effects of the medication.

### 2.3. Ethical Considerations

The present study was approved by the Research Ethics Committee of FIOCRUZ-PE, Recife, PE, Brazil (CEP-FIOCRUZ/PE, 41/2008).

## 3. Discussion

“Occupational disease” defines a change in a worker's health caused by chemical, environmental, biological, psychological, or other work-related factors. Cutaneous leishmaniasis and mucocutaneous leishmaniasis are listed as occupational diseases by the Brazilian Health Ministry [13]. Agricultural and forestry work in endemic areas and other specific situations, such as deforestation work for building roads, fieldwork for research biologists and military training are examples of situations with a risk of occupational exposure to *Leishmania braziliensis* [14]. Laboratory or nosocomial environment work-related accidents with *Leishmania* spp. involve percutaneous accidental contamination while handling fluids, culture, and/or laboratory animals [2].

In this paper, we report two different occupationally acquired ACL cases: one accidental laboratory contamination while handling an infected sample and one infection acquired during field studies unrelated to ACL.

Linking ACL to occupational accidents may be impaired by a lack of epidemiological ground knowledge, its similarity to other dermatological injures, or coinfection with other pathogens [15]. Association with fungi and bacteria is common in ACL lesions and constitutes a complicating factor for the clinical diagnosis and treatment of leishmaniasis.

For example, strains of *A. baumannii* and *E. faecalis* were identified by culture in the ACL lesion in Case 1. However, they were excluded as causative agents because tigecycline administration failed to heal the lesion. Response to the antimonial therapy is an auxiliary ACL diagnosis criterion, and the two cases were successfully healed in this way.

Although several *Leishmania *species are in circulation in Brazil, *L*. (*V*.) *braziliensis* is the most medically important species; it is distributed nationwide and causes a more severe form of the disease than do the other circulating species [3, 15]. *L*. (*V.*) *naiffi* is alleged to produce a mild infection in humans, and its spread is restricted to the Pará and Amazonas states [16].

Early and accurate ACL diagnosis is essential for rapid and effective treatment. PCR-based procedures can distinguish *Leishmania* species and subspecies in samples from different sources and have proved useful for confirming the persistence of the parasite in scars [17–21].

A persistence of the parasite in spite of lesion healing occurred in Case 1, and the patient developed a new lesion one month after completion of the first antimonial treatment cycle. Complete clinical cure was achieved at end of a second treatment cycle.

In our work, the combination of the swab sample collection technique and PCR proved to be an efficient means for ACL diagnosis in the two patients. Implementation of the swab method for routine sample collection is feasible and has many advantages: it can be performed by any health-care, nonphysician professional; it does not require anesthesia; the procedure can be carried out at the patient's home or in an outpatient setting, without the discomfort of patient displacement and hospitalization.

The swab technique allows collection from the full extent of the lesion. Unlike invasive methods such as tissue biopsy, swab collection minimizes the risk of iatrogenic infections that may compromise diagnosis and patient recovery [8]. Furthermore, because it does not use any “sharps” devices, this method is safer for the professionals involved on biological material collection.

In conclusion, this paper illustrates the need for individual protective procedures by health care professionals dealing with ACL patients. The swab method for wound sample collection would reduce the risk of accidental infection during the collecting and handling of diagnostic samples. The importance of the proper use of personal protective equipment (PPE) by exposed workers should be duly emphasized and its use should be required, both in clinical settings and in field or laboratory activities.
